# Two cases of direct aortic transcatheter aortic valve implantation via anterior right Mini-thoracotomy after preoperative virtual reality-based simulation: a case report

**DOI:** 10.1093/ehjcr/ytaf203

**Published:** 2025-04-25

**Authors:** Chiaki Aichi, Masanori Yamamoto, Hirooki Higami, Hideki Kitamura

**Affiliations:** Department of Cardiovascular Surgery, Nagoya Heart Center, 1-1-14 Sunadabashi, Higashi-ku, Nagoya, Aichi Prefecture 461-0045, Japan; Department of Cardiovascular Medicine, Toyohashi Heart Center, 21-1 Oyamacho, Gofunotori, Toyohashi, Aichi Prefecture 441-8530, Japan; Department of Cardiovascular Medicine, Gifu Heart Center, 4-14-4 Yabutaminami, Gifu, Gifu Prefecture 500-8384, Japan; Department of Cardiovascular Surgery, Nagoya Heart Center, 1-1-14 Sunadabashi, Higashi-ku, Nagoya, Aichi Prefecture 461-0045, Japan

**Keywords:** Aortic stenosis therapy, Anterior right mini-thoracotomy, Direct aortic transcatheter aortic valve implantation, Minimally invasive cardiac surgery, Virtual reality, Case report

## Abstract

**Background:**

In direct aortic transcatheter aortic valve implantation for patients with aortic stenosis, anterior right mini-thoracotomy presents challenges due to the limited visual field and raises concerns about significant bleeding. Furthermore, identifying the site closest to the ascending aorta while maintaining an adequate distance from the annulus is challenging. We report on the effectiveness of our preoperative simulation using virtual reality technology to ensure a safe and precise direct aortic transcatheter aortic valve implantation via anterior right mini-thoracotomy in two patients.

**Case summary:**

The patients were an 81-year-old man (Patient 1) and an 86-year-old woman (Patient 2) with aortic stenosis; their ascending aorta was closest to the third intercostal space. In Patient 1, virtual reality simulation revealed that, according to the distance and angulation from the annulus, the ideal puncture site was the second intercostal space. Accordingly, an anterior right mini-thoracotomy was performed at the second intercostal space, and a 23-mm SAPIEN 3 valve (Edwards Lifesciences Ltd.) was adequately implanted. Conversely, in Patient 2, the virtual reality assessment indicated that the third intercostal space was the appropriate site of entry. Therefore, anterior right mini-thoracotomy was performed at the third intercostal space, and a 23-mm SAPIEN 3 valve was successfully implanted, without difficulties.

**Discussion:**

The use of preoperative virtual reality simulation enabled the safe and precise execution of direct aortic transcatheter aortic valve implantation via anterior right mini-thoracotomy. Virtual reality technology is expected to have broader applications in cardiac surgery in the future.

**Learning points**

We performed direct aortic transcatheter aortic valve implantation via anterior right mini-thoracotomy in two cases using virtual reality (VR) simulation.

The anatomy from the aortic annulus to the ascending aorta varies greatly between individuals; however, with VR simulation, both cases were safely completed.

## Introduction

In cases where peripheral access is difficult for transcatheter aortic valve implantation (TAVI), direct aortic TAVI (DA-TAVI) is selected. Among DA-TAVI approaches, anterior right mini-thoracotomy (ART) is a less invasive approach, as it does not require a sternotomy and provides a favourable angle for access to the annulus.^1^ However, compared to sternotomy, ART presents challenges due to its narrower surgical field and the greater distance from the incision site to the aortic puncture site. Additionally, the puncture site must be at least 5.5–6.0 cm away from the annulus for adequate valve alignment. To address this issue, we performed preoperative three-dimensional measurements using virtual reality (VR) technology. VR is a three-dimensional virtual environment artificially constructed using computer technology, in which users can visually and intuitively manipulate data such as anatomical models. It has been reported that VR-based simulation is useful for customizing guiding catheters for coronary interventions^2^ and delivery sheaths for percutaneous left atrial appendage closure.^3^ However, no studies to date have demonstrated the application and usefulness of VR technology in DA-TAVI. In this paper, we report the effectiveness of pre-procedural VR simulation for the safety and precision of DA-TAVI via ART in two patients.

## Summary figure

We performed transcatheter aortic valve implantation (TAVI) via right mini-thoracotomy in two cases using virtual reality (VR) simulation. In both cases, the exact distance from the puncture site to the valve annulus was measured. VR simulation enabled the safe and precise execution of direct aortic TAVI.

**Figure:**
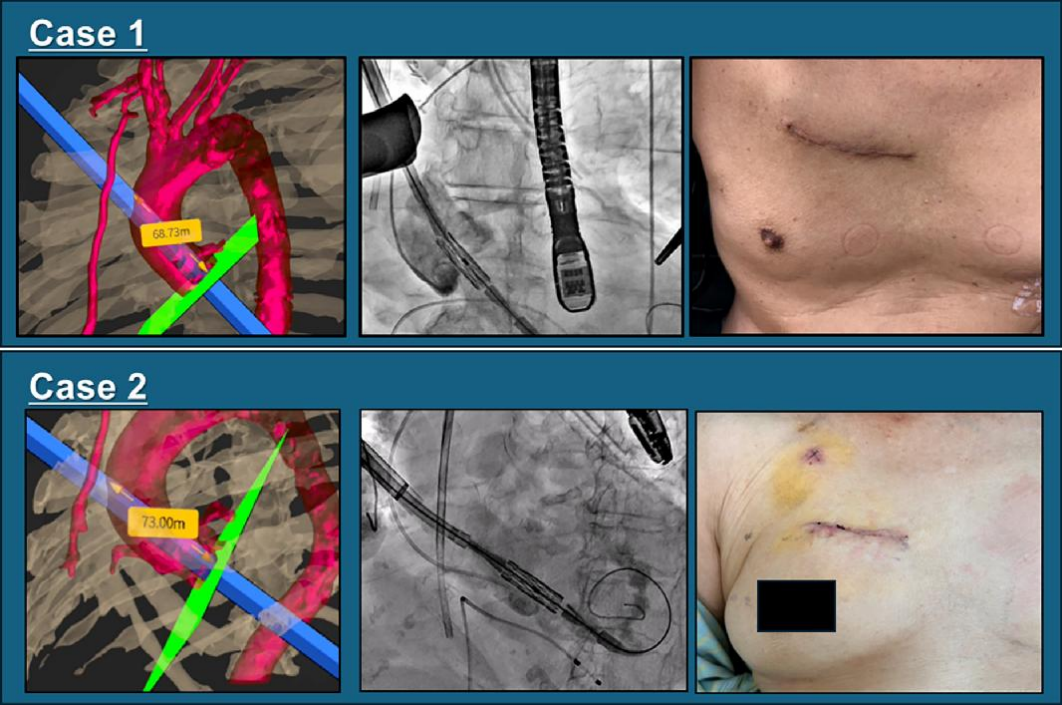


## Patient 1

**Figure 1:**
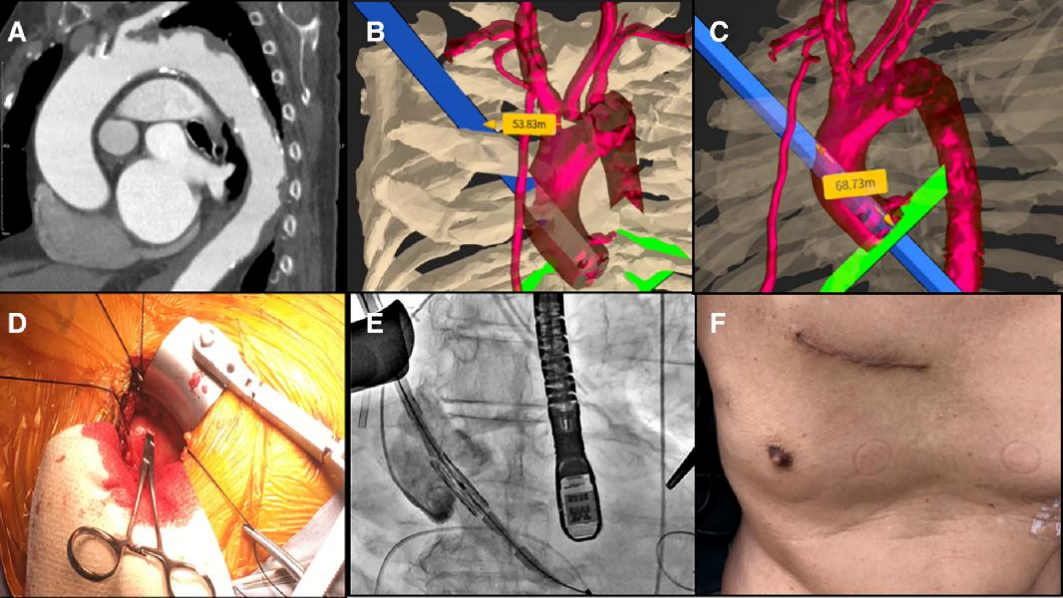
(*A*) An 81-year-old male underwent direct aortic TAVI due to plaques in the aortic arch. (*B* and *C*) VR simulation identified the second intercostal space, 53 mm from the right sternal edge, with a 68-mm distance to the annulus. (*D* and *E*) A rib retractor was used to expose the aorta, and the valve was implanted. (*F*) The patient was discharged on postoperative day 4.

An 81-year-old man with severe aortic stenosis and exertional dyspnoea was scheduled for TAVI. Preoperative blood tests, electrocardiography, and pulmonary function tests revealed no significant abnormalities, while contrast-enhanced computed tomography demonstrated the presence of plaques in the distal aortic arch, necessitating DA-TAVI. VR simulation identified the third intercostal space as closest to the ascending aorta but insufficient in distance to the annulus. The incision site, 53 mm from the right sternal edge, provided a confirmed 68-mm distance to the annulus. Under general anaesthesia with one-lung ventilation, an intercostal nerve block with ropivacaine was performed, and a 5-cm thoracotomy was made at the third intercostal space using a wound protector instead of a rib retractor. A purse-string suture was placed, and a 23-mm SAPIEN 3 Ultra RESILIA valve (Edwards Lifesciences Ltd., Irvine, CA, USA) was implanted. Postoperative echocardiography showed no aortic regurgitation and a mean pressure gradient of 14 mmHg. The patient was discharged on postoperative day 4. (*Figure 1*). It has now been approximately one year since the surgery, and the patient is doing well at home without any incisional pain.

## Patient 2

**Figure 2:**
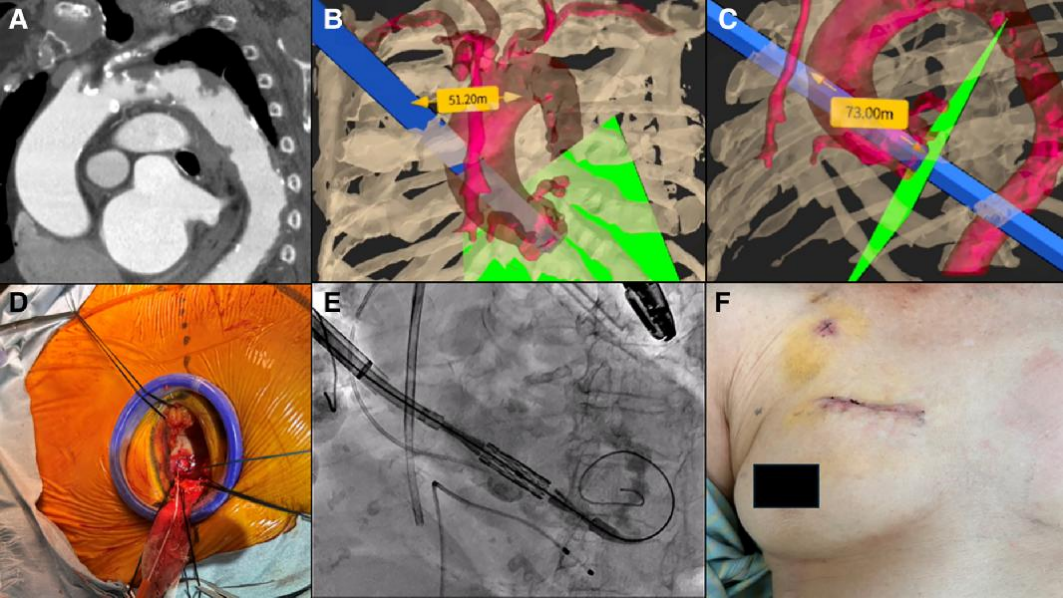
(*A*) An 86-year-old female underwent direct aortic TAVI due to plaques in the aortic arch. (*B* and *C*) VR simulation identified the third intercostal space, 51 mm from the right sternal edge, with a 73-mm distance to the annulus. (*D* and *E*) A wound protector was used to expose the aorta, and the valve was implanted. (*F*) The patient was discharged on postoperative day 10.

An 86-year-old woman with severe aortic stenosis and exertional dyspnoea was scheduled for TAVI. Preoperative blood tests, electrocardiography, and pulmonary function tests revealed no remarkable abnormalities. However, contrast-enhanced computed tomography identified a shaggy aorta and a thrombus in the subclavian artery, leading to the decision to perform DA-TAVI. VR simulation revealed the third intercostal space as closest to the ascending aorta with an adequate distance to the annulus for ART and a suitable device delivery angle. The incision site was 51 mm from the right sternal edge, with a 73-mm distance to the annulus. Under general anaesthesia with one-lung ventilation, an intercostal nerve block using ropivacaine was performed, followed by a 5-cm thoracotomy at the third intercostal space with a wound protector instead of a rib retractor. The distance to the annulus was confirmed at 78 mm. A 23-mm SAPIEN 3 Ultra RESILIA valve was implanted. Postoperative echocardiography showed trivial aortic regurgitation and a mean pressure gradient of 11 mmHg. The patient was discharged on postoperative day 8. (*Figure 2*). It has been about 6 months since the surgery, and the patient is living comfortably at home without any discomfort at the incision site.

## Discussion

In this paper, we report on the application and usefulness of VR technology in DA-TAVI via ART in two patients. The application we used allows for the creation of three-dimensional models of the ribs and aorta from preoperative cardiac computed tomography data, which were then reproduced in a virtual space. This allowed us to predict the optimal location for the ART incision accurately, including the particular intercostal space and the distance from the sternum. Additionally, we could measure the angle between the puncture site and the annulus. For instance, in Patient 2, the planned puncture site on the ascending aorta was predicted to be very close to the skin; thus, the skin incision was made smaller than that in Patient 1. Additionally, in Patient 2, the ascending aorta had a horizontal orientation, commonly termed a ‘horizontal aorta’. By deliberately selecting a lower intercostal space, we could achieve an appropriate angle for device delivery. As predicted during preoperative simulation, the ascending aorta was clearly visible from this position, allowing the device to be positioned perpendicularly to the annulus. In both patients, the procedure was completed using only the main incision, and no additional skin incisions were needed for sheath insertion.^4^

Our experience with these cases demonstrated that preoperative planning using VR offers significant advantages over computed tomography-based planning. Unlike 3D reconstruction with volume rendering, VR allows free manipulation and observation of a life-sized three-dimensional heart model, enabling more accurate distance calculations. Furthermore, in the virtual space, the immersive experience creates the illusion that the structures are physically present, allowing surgeons to intuitively perceive the distance from the ribs to the aorta and visualize the surgical procedure in greater detail (see Supplementary material online, *Video S1*).

The thickness of the chest and the position of the aortic valve vary greatly among individuals. Therefore, currently, we are developing the application of VR simulation for minimally invasive cardiac surgery to reproduce the placement of the main incision and ports preoperatively. In the future, the application of this technology in cardiac surgery is likely to be highly useful, particularly for surgeons in training.

## Supplementary Material

ytaf203_Supplementary_Data

## Data Availability

The data underlying this article are available to use for all readers.

## Lead author biography

Chiaki Aichi, a cardiovascular surgeon at the Nagoya Heart Center in Aichi, Japan, specializes in minimally invasive cardiac surgery and structural heart disease.

## Notes

**Funding:** This study has received no financial support.
